# TM9SF4 acts as a receptor mediating *Glaesserella parasuis* cytolethal distending toxin–induced cytotoxicity in PK15 cells

**DOI:** 10.3389/fcimb.2026.1783709

**Published:** 2026-03-19

**Authors:** Li Lei, Shiyu Xu, Zhen Yang, Xingyu Pan, Jiali Xu, Senyan Du, Qin Zhao, Xiaobo Huang, Sanjie Cao, Rui Wu, Yiping Wang, Qigui Yan, Yiping Wen

**Affiliations:** 1 Research Center for Swine Diseases, College of Veterinary Medicine, Sichuan Agricultural University, Chengdu, China; 2 Institute of Urban Agriculture, Chinese Academy of Agricultural Sciences, Chengdu Agricultural Science and Technology Center, Chengdu, China; 3 Chongqing Academy of Animal Sciences, Chongqing, China

**Keywords:** *Glaesserella parasuis*, Cytolethal Distending Toxin, interaction proteins, cell receptor, TM9SF4

## Abstract

**Background:**

Cytolethal Distending Toxin (CDT) is the only exotoxin that *Glaesserella parasuis* (*G. parasuis*) can secrete. *G. parasuis* CDT (*Gp*CDT) triggers DNA damage responses, leading to irreversible cell cycle arrest and apoptosis, playing an important role in the pathogenic process of *G. parasuis*. Currently, research on the host cell receptors of *Gp*CDT remains limited. Screening and identification of host cell receptors that interact with *Gp*CDT are crucial for systematically elucidating the cytotoxic mechanisms induced by this toxin.

**Methods:**

This study employed Co-immunoprecipitation (Co-IP) combined with Liquid Chromatography-Tandem Mass Spectrometry (LC-MS/MS) to identify potential host proteins interacting with *Gp*CDT in PK15 cells. Nine Proteins were selected for further evaluation based on subcellular localization and Gene Ontology classification. Eukaryotic expression and Co-IP validated four interacting proteins. Subsequently, heterozygous knockout PK15 cell lines for these genes were generated via CRISPR/Cas9, and CCK-8 assays identified TM9SF4 as having the most significant impact on *Gp*CDT virulence. Therefore, a homozygous *TM9SF4* knockout PK15 cell line (KO) was generated via limited dilution, and a stable *TM9SF4*-overexpressing PK15 cell line (OE) was established through lentiviral packaging. Western blotting and qRT-PCR confirmed protein and gene expression, and CCK-8 assays combined with cytopathic effect (CPE) observation determined the role of TM9SF4 in *Gp*CDT-induced cytotoxicity. Finally, indirect immunofluorescence was performed to assess co-localization of TM9SF4 with *Gp*CDT.

**Results:**

We identified 287 proteins in PK15 cells that potentially interact with *Gp*CDT, among which 58 were localized to the plasma membrane or extracellular. Nine proteins were selected for further investigation. Among them, EPHB4, LITAF, TM9SF4, SLC12A4 interacted with *Gp*CDT, but only the deficiency of TM9SF4 significantly inhibited the virulence of the *Gp*CDT. Results from CCK-8 and CPE showed that KO cells exhibited significantly higher survival rates and suppressed *Gp*CDT-induced cellular distention and cell death, whereas OE cells showed decreased survival rates and typical cytopathic change. Finally, indirect immunofluorescence confirmed strong co-localization between TM9SF4 and *Gp*CDT.

**Conclusion:**

We initially proposed TM9SF4 as a receptor for *Gp*CDT in PK15 cells, essential for *Gp*CDT binding and cytotoxicity. This study may provide a new theoretical basis for targeted prevention and treatment of swine Glässer’s disease.

## 1 Introduction

*Glaesserella parasuis* (*G. parasuis*) is a Gram-negative conditional pathogen that colonizes the upper respiratory tract of pigs (Møller and Kilian, 1990). It can cause swine Glässer’s disease, which is characterized by fibrinous polyserositis, polyarthritis, and meningitis (Vahle et al., 1995). Currently, vaccination and antibiotics are the mainstays for preventing and controlling Glässer’s disease. However, the limited cross-protection offered by vaccines, coupled with rising antibiotic resistance, presents a serious challenge to its effective management (Costa-Hurtado et al., 2020). Therefore, deciphering the pathogenic mechanisms of *G. parasuis* virulence factors is crucial for developing novel strategies to prevent and control swine Glässer’s disease.

Cytolethal Distending Toxin (CDT) is a heat-labile bacterial genotoxin that was first discovered in the culture filtrates of *Escherichia coli* in the late 1980s (Johnson and Lior, 1988). CDT is encoded by three genes, *cdtA*, *cdtB*, and *cdtC*, which specify three distinct polypeptides, these subunits assemble into a functional heterotrimeric holotoxin (Jinadasa et al., 2011). It is produced by various Gram-negative microaerophilic bacteria, including *Glaesserella parasuis*, *Escherichia coli*, *Aggregatibacter actinomycetemcomitans*, *Campylobacter jejuni*, and *Haemophilus ducreyi*, among others. CDT is a nuclear-targeting bacterial toxin. Upon binding to plasma membrane receptors, it undergoes clathrin- and dynamin-dependent endocytosis and subsequently retrograde transport from early endosomes or late endosomes to the Golgi and then on to the endoplasmic reticulum (ER) before final translocation into the nucleus (Guerra et al., 2011; Martina et al., 2017; Du and Song, 2022; Zhang et al., 2024). In addition to its unique DNase I activity, which induces double-strand breaks in mammalian cell DNA, leading to irreversible cell cycle arrest and apoptosis, CDT also triggers pathological effects such as cellular distention, actin cytoskeleton remodeling, and nuclear enlargement (Li et al., 2017; Huerta-Cantillo et al., 2025).

The binding of CDT to plasma membrane receptors is a prerequisite for its toxicity. However, the identity of these cellular surface receptors remains unclear and controversial. For instance, N-linked glycoproteins have been reported to serve as receptors for *E. coli* CDT (*Ec*CDT), *A. actinomycetemcomitans* CDT (*Aa*CDT), and *H. ducreyi* CDT (*Hd*CDT) (Cao et al., 2005; McSweeney and Dreyfus, 2005; Eshraghi et al., 2010). Supporting this, inhibition of N-linked glycan synthesis with tunicamycin in CHO-K1 cells reduced cellular sensitivity to *Ec*CDT, *Aa*CDT, and *Hd*CDT. In contrast, tunicamycin treatment did not protect cells from *C. jejuni* CDT (*Cj*CDT), suggesting that glycoproteins may not function as receptors for *Cj*CDT (Eshraghi et al., 2010). In another research, glycosphingolipids (GSLs) including GM1, GM2, GM3 and Gb4 have also been identified as binding partners for *Aa*CDT (Mise et al., 2005). This is supported by the finding that PPMP, a glucosylceramide synthesis inhibitor causing reversible GSL depletion, reduces *Aa*CDT intoxication in human monocyte cell line U937, with GM3 showing the most significant inhibitory effect on toxicity (Mise et al., 2005). Furthermore, Boesze-Battaglia et al. used laser confocal microscopy to demonstrate that *Aa*CDT co-localizes with GM1-enriched membrane domains in the plasma membrane (Boesze-Battaglia et al., 2006). Additionally, a haploid genetic screen identified several host factors associated with CDT binding. For instance, the G-protein coupled receptor homolog (TMEM181) is closely linked to *Ec*CDT binding, *Aa*CDT cellular binding depends on synaptogyrin 2 (SYNGR2), and *Cj*CDT relies on transmembrane protein 127 (TMEM127) and an uncharacterized G-protein coupled receptor (GPR107) (Carette et al., 2011). Collectively, these findings suggest that the receptors for species-specific CDTs may differ across host cells.

*G. parasuis* CDT (*Gp*CDT), the only known exotoxin secreted by *Glaesserella parasuis*, plays a significant role in swine Glässer’s disease pathogenesis. However, current research on *Gp*CDT has primarily focused on its intracellular trafficking and cytotoxic effects. Studies show that *Gp*CDT employs GTPase 4b (Rab4b) for early endosomal transport (Zhang et al., 2024). It also compromises epithelial barrier function by modulating the expression of the tight junction protein occludin (OCLN) in newborn pig tracheal (NPTr) epithelial cells, thereby promoting infection (Yang et al., 2023). Furthermore, *Gp*CDT induces cellular distention, cell cycle arrest, and p53-dependent apoptosis in porcine kidney epithelial (PK15) cells and porcine alveolar macrophages (PAM), as well as triggering ferroptosis through disruption of iron metabolism and redox homeostasis (Li et al., 2017; Xu et al., 2025). In contrast, the host cell receptor for *Gp*CDT remains uncharacterized.

Therefore, this study aimed to identify host proteins in PK15 cells that interact with *Gp*CDT. Using Co-IP combined with LC-MS/MS, we selected nine candidate receptors based on subcellular localization and Gene Ontology classification for further investigation. Through a series of assays, including the Co-IP, knockout cell lines construction, cell viability (CCK-8), and IFA, we demonstrated that TM9SF4 specifically interacts with *Gp*CDT and is essential for mediating *Gp*CDT cytotoxicity. These findings indicate that TM9SF4 functions as a receptor for *Gp*CDT. Our work lays the groundwork for further investigation into the specific role of TM9SF4 in *Gp*CDT cytotoxicity, and provides a novel theoretical basis for developing targeted strategies against swine Glässer’s disease.

## 2 Materials and methods

### 2.1 Cells, plasmids and bacterial strains

PK15 and HEK-293 T cells were preserved by the Swine Disease Research Center of the College of Veterinary Medicine, Sichuan Agricultural University and grown in Dulbecco’s modified Eagle’s medium (DMEM) (Gibco, USA) supplemented with 10% fetal bovine serum (FBS) (Gibco, USA), incubated at 37 °C in a 5% CO_2_ humidified atmosphere.

LentiCRISPR v2 (52961), pMD2.G (12259), psPAX (12260) and pCDH-CMV-MCS-EF1-copGFP-T2A-Puro (72263) were purchased from Addgene. pcDNA3.1 (+)-C-Myc (ZB56893) were purchased from Sangon Biotech. The recombinant plasmids pET-32a-his_6_-*cdtA*, pET-32a-his_6_-*cdtB*, pET-32a-his_6_-*cdtC* were made in our laboratory.

E. coli DH5α and Rosetta (DE3) (TransGene Biotech, China) were cultured in Luria-Bertani (LB) (Hopebio, China) medium.

### 2.2 Expression and purification of *Gp*CDT

The pET-32a-his_6_-*cdtA*, pET-32a-his_6_-*cdtB*, pET-32a-his_6_-*cdtC* plasmids were constructed and preserved in our laboratory and were transformed into Rosetta (DE3) pLysS. The transformed cells were cultured in LB medium supplemented with ampicillin (AMP^+^) until the optical density at 600 nm (OD_600_) reached 0.8 - 1.0. Then, recombinant clones were induced with 0.2 mM IPTG (Beyotime, China) for 12 hours at 25 °C (CdtA-His_6_, CdtC-His_6_) or 18°C (CdtB-His_6_). Subsequently, the three fusion recombinant proteins with His-tags were purified by Ni affinity chromatography (Bio-Rad, USA). Thereafter, CdtA-His_6_, CdtB-His_6_, and CdtC-His_6_ were mixed in a molar ratio of 1:1:1 to prepare the CDT holotoxin (*Gp*CDT). After being gently mixed at 4 °C for 2 hours, the protein mixture was transferred into a dialysis bag (10 kDa MWCO, Solarbio, China). The mixture was then dialyzed against PBS buffer for 36 hours, and the dialysis buffer was refreshed every 12 hours. In addition, the purification protocol of pET-32a His-tagged protein is similar to that of CDT subunits (Yang et al., 2023).

### 2.3 Sample preparation for LC-MS/MS

The PK15 cell membrane protein was extracted according to the operation instructions of the cell membrane protein extraction kit (Proteintech, China). For every 100 µl of membrane protein, add 20 µg of *Gp*CDT holotoxin protein or control protein (His-tagged protein), and co-incubate at 4 °C for 12 hours. Protein A/G Magnetic Beads (MCE, China) resuspend and washed three times with cold PBST, then add Mouse anti-his-tag mAb (1:50) (ABclonal, China) with cold PBS, incubated at 4°C for 12 h. Subsequently, wash the beads with cold PBST three times. Then, add the premixed *Gp*CDT protein–membrane protein or His-tagged protein–membrane protein mixture to the beads and incubate at 4 °C for 12 hours. After the incubation was completed, the beads were eluted and sent to PTM BIO Company for further testing. First, the sample pH was adjusted to alkaline conditions. A 5 μL aliquot of the suspension was mixed with loading buffer, boiled, and subjected to silver staining for detection. Subsequently, equal amounts of protein from each sample were taken for digestion. The volume was adjusted with lysis buffer, and dithiothreitol (DTT) was added to a final concentration of 5 mM, followed by reduction at 56 °C for 30 min. Iodoacetamide (IAA) was then added to a final concentration of 11 mM, and the mixture was incubated at room temperature in the dark for 15 min. TEAB was added to dilute the urea to a concentration below 2 M. Trypsin was added at a 1:50 (enzyme:protein, m/m) ratio for overnight digestion, followed by a second addition of trypsin at a 1:100 ratio for an additional 4 h digestion.

### 2.4 Liquid chromatography-tandem mass spectrometry analysis

The peptides were first dissolved in mobile phase A and then separated using a Vanquish Neo ultra-high performance liquid chromatography (UHPLC) system. Mobile phase A consisted of an aqueous solution containing 0.1% formic acid and 2% acetonitrile, while mobile phase B was composed of 0.1% formic acid and 90% acetonitrile in water. The liquid chromatography gradient was programmed as follows: 0–22.5 min, 6%–22% B; 22.5–26.5 min, 22%–34% B; 26.5–28.5 min, 34%–80% B; and 28.5–30 min, held at 80% B. The flow rate was maintained at 700 nL/min throughout the separation. After separation by the UHPLC system, the peptides were injected into an NSI ion source for ionization and subsequently analyzed using an Orbitrap Exploris 480 mass spectrometer. The ion spray voltage was set at 2300 V, and the FAIMS compensation voltage (CV) was set at –45 V. Both precursor ions and their corresponding fragment ions were detected and analyzed using the high-resolution Orbitrap mass analyzer. The full MS scan range was set from 350 to 1400 m/z with a resolution of 60,000. For MS/MS analysis, the scan range was fixed with a starting point of 120 m/z, and the resolution was set to 15,000. Data acquisition was performed using a data-independent acquisition (DIA) mode. In this mode, following each full MS scan, peptide ions within consecutive m/z windows were sequentially selected and fragmented in the HCD collision cell using a normalized collision energy of 27%, followed by MS/MS analysis. To optimize the efficiency of mass spectrometry utilization, the automatic gain control (AGC) target was set to 1E6, and the maximum injection time was set to 22 ms.

### 2.5 Database search

The DIA data obtained in this study were processed using the DIA-NN (v 1.8) search engine with default parameters. The spectral library was generated using a protein database (XB03060DA.fasta, containing 60,746 sequences). Digestion was set to Trypsin/P, allowing up to one missed cleavage. Fixed modifications included N-terminal methionine excision and cysteine carbamidomethylation. A deep learning-based algorithm was employed to generate a predicted spectral library, and a decoy database was included to estimate the false discovery rate (FDR) resulting from random matches. The FDR for precursor identification was set to 1%.

### 2.6 Bioinformatics analysis

The identified proteins were functionally annotated using commonly used databases, including Gene Ontology (GO) and Kyoto Encyclopedia of Genes and Genomes (KEGG). Based on quantitative results, fold changes (FC) between groups and p-values from t-tests were calculated. Differentially expressed proteins were screened according to defined thresholds, and corresponding statistical plots were generated. Subsequently, functional classification analyses were performed on the differentially expressed proteins between groups, including GO secondary classification, subcellular localization prediction, COG/KOG functional categorization, and KEGG pathway enrichment. This study utilized the UniProt (https://www.uniprot.org/) and Gene Ontology (GO) (https://www.geneontology.org/) databases, combined with subcellular localization information, to analyze the mass spectrometry data and thereby identify a series of candidate proteins that interact with *Gp*CDT.

### 2.7 Eukaryotic expression in HEK-293T cells

In light of the subcellular localization of proteins and GO functional analysis, nine proteins were selected for subsequent investigation. According to the coding DNA sequence (CDs) of genes in the National Center for Biotechnology Information (NCBI), namely EPHB4 (XM_003124371.5), ITGA5 (XM_001925252.7), APP (NM_214372.1), UGCG (XM_001925267.7), GOLGA7 (XM_005672664.3), LITAF (XM_021086604.1), TM9SF4 (XM_005672825.3), SLC12A4 (NM_213949.2) and SEC63 (XM_001925479.7), primers were designed by employing the double digestion method and the pcDNA3.1(+)-C-Myc plasmid. These primers were synthesized by Sangon Biotech Company. All primers are listed in **Table 1**.

**Table 1:** Primers used in this study.

Primers	Sequence (5’—3’)	Size (bp)
EPHB4-F	CTAGCGTTTAAACTTAAGCTTATGGAGCTCCGGGCTCTG	3003
EPHB4-R	TGCTGGATATCTGCAGAATTCGTACTGGGGGGCTGGTGC	
ITGA5-F	CTAGCGTTTAAACTTAAGCTTATGGGGAGCCGGACGCCA	3213
ITGA5-R	TTCGGATCCGAGCTCGGTACCGGCATCAGAGGTGGCTGGA	
APP-F	CTAGCGTTTAAACTTAAGCTTATGCTGCCCGGTTTGGCA	2352
APP-R	TGCTGGATATCTGCAGAATTCGTTCTGCATCTGCTCAAAGAACTT	
UGCG-F	CTTGGTACCGAGCTCGGATCCATGGCGCTGCTGGACCTG	1224
UGCG-R	CTCGGGCCCTCTAGACTCGAGTACATCCAGGATCTCCTCGGC	
GOLGA7-F	CTAGCGTTTAAACTTAAGCTTATGAGGCCGCAGCAGGCGCCG	453
GOLGA7-R	TGCTGGATATCTGCAGAATTCTCTCCCACTGCTCACGCCTCTGTCTTCAT	
LITAF-F	CTAGCGTTTAAACTTAAGCTTATGTCTGCTCCAGGAGCCTACC	525
LITAF-R	TGCTGGATATCTGCAGAATTCCAAACGCTTGTAGGTGCCCA	
TM9SF4-F	CCAAGCTGGCTAGTTAAGCTTATGAACAGTGAAAAGAAGTGTGAGGT	1691
TM9SF4-R	TGCTGGATATCTGCAGAATTCCGGTCTATCTTCACAGCAGCGTAGATCT	
SLC12A4-F	CTAGCGTTTAAACTTAAGCTTATGCCTCACTTCACCGTGGTG	3300
SLC12A4-R	TGCTGGATATCTGCAGAATTCGGAGTAGATGGTGATGACTTCCCG	
SEC63-F	ACCGAGCTCGGATCCGAATTCATGTTCATGAGAATAGCAAAAGCTTAT	1902
SEC63-R	CTCGGGCCCTCTAGACTCGAGGTCATCATCCTCCTCTTCTTCTTCC	
sgRNA-EPHB4-F	CACCGAACCCCTACATCAAGGTACCC	
sgRNA-EPHB4-R	AAACGGGTACCTTGATGTAGGGGTTC	
sgRNA-LITAF-F	CACCGAGGCCGTACATACTTGCGT	
sgRNA-LITAF-R	AAACACGCAAGTATGTACGGCCTC	
sgRNA-TM9SF4-F	CACCGTCGTGGCCGAGCGAATCACAG	
sgRNA-TM9SF4-R	AAACCTGTGATTCGCTCGGCCACGAC	
sgRNA-SLC12A4-F	CACCGCGAGGTTGTCATAGTCGCCG	
sgRNA-SLC12A4-R	AAACCGGCGACTATGACAACCTCGC	
EPHB4-KO-F	AGGAGACCTTCACCGTCTTCTA	161
EPHB4-KO-R	GCTGCTTCTAGACCACAGGC	
LITAF-KO-F	TAAACAGTTACTACCCCACGCC	251
LITAF-KO-R	ACTGAGCTGACTGGCTTAGACC	
TM9SF4-KO-F	AAAAGAAGTGTGAGGTTCTGTGC	287
TM9SF4-KO-R	TTTCTCTTTCTTCTTGTCATCGC	
SLC12A4-KO-F	GCGATGCCTCACTTCACC	272
SLC12A4-KO-R	ACACACTGACTGAACGAAGGC	
TM9SF4-OE-F	GATTCTAGAGCTAGCGAATTCATGAACAGTGAAAAGAAGTGTGAGGTTCTG	1693
TM9SF4-OE-R	GATCGCAGATCCTTCGCGGCCGCGCGTCTATCTTCACAGCAGCGTAGATCT	
qTM9SF4-F	AACAAGCCGGTGACCCTGAC	98
qTM9SF4-R	CAGGTTGTCCGCAATGAGGTGG	
β-actin-F	CCACGAAACTACCTTCAACTCC	132
β-actin-R	GTGATCTCCTTCTGCATCCTGT	
GAPDH-F	GGCGTGAACCACGAGAAGTATAA	119
GAPDH-R	CCCTCCACGATGCCAAAGTG	

The CDs regions of the target genes were amplified using the cDNA of PK15 cells as a template. Following purification and recovery of the amplified products, they were ligated into linear vectors to generate recombinant plasmids. The recombinant plasmids were confirmed by sequencing. The constructed recombinant plasmids and empty plasmids were transiently transfected into HEK-293T cells using Lipofectamine 3000 and P3000 transfection reagents (Invitrogen, USA). After 48 hours of transfection, total proteins were harvested by Nondenaturing lysis Buffer (Solarbio, China). Successful expression of the target proteins was confirmed by western blotting.

### 2.8 Co-immunoprecipitation

Pre-washed Protein A/G Magnetic Beads (50 μL) were resuspended in 200 μL cold PBS along with either mouse anti-His-Tag mAb (1:100, ABclonal, China) or rabbit anti-Myc-tag pAb (1:200, HUABIO, China). The mixture was incubated at 4 °C for 2 h with gentle rotation. The beads were then washed three times with cold PBST. Subsequently, 2 μg *Gp*CDT-His or 200 μL whole-cell lysate containing the individually expressed eukaryotic protein (EPHB4, ITGA5, APP, UGCG, GOLGA7, LITAF, TM9SF4, SLC12A4, SEC63) was added and incubated with the beads at 4 °C for another 2 h, followed by three washes with cold PBST. Next, the complementary binding partner (either the eukaryotic whole-cell lysate or *Gp*CDT-His) was added to the beads, and the mixture was incubated at 4 °C for 2 h. After incubation, the beads were stringently washed seven times with cold PBST. Finally, the bound complexes were eluted by boiling in SDS-PAGE loading buffer for 5 minutes. The eluted proteins were analyzed by Western blotting to detect the interaction between the eukaryotic protein and *Gp*CDT.

### 2.9 Gene polyclonal knockout by CRISPR/Cas9

Co-IP confirmed that EPHB4, LITAF, TM9SF4 and SLC12A4 interact with the *Gp*CDT protein. To further investigate these interactions, CRISPR/Cas9 was employed to generate polyclonal knockout PK15 cell lines. sgRNA sequences targeting each of the four genes were designed using the CHOPCHOP online tool (https://chopchop.cbu.uib.no/). Namely EPHB4-sgRNA (AACCCCTACATCAAGGTACCCAGG), LITAF-sgRNA (GAGGCCGTACATACTTGCGTTGG), TM9SF4-sgRNA (TCGTGGCCGAGCGAATCACAGAGG), SLC12A4-sgRNA (CGAGGTTGTCATAGTCGCCGCGG). Above oligonucleotides sgRNA were annealed via touchdown PCR to form double-stranded structures and ligated into the lentiCRISPR v2 vector.

The recombinant lentiCRISPR-v2-sgRNA plasmid was then co-transfected with the packaging plasmids psPAX and pMD2.G into HEK-293T cells using Lipofectamine 3000. The supernatants containing lentiviral particles were collected 48h later. PK15 cells were exposured to the filtered (0.22μM) lentiviral supernatant for 48 hours, and cultured in fresh medium with 5 μg/mL puromycin (Beyotime, China) to select positive cells. Successful polyclonal knockout PK15 cell lines were confirmed by sequencing and named PK15*ΔEPHB4*^+/-^, PK15*ΔLITAF*^+/-^, PK15*ΔTM9SF4*^+/-^, PK15*ΔSLC12A4*^+/-^.

### 2.10 Screening of *TM9SF4* monoclonal knockout PK15 cell lines

The homozygous knockout cell line of the *TM9SF4* gene was screened using the limiting dilution method. The PK15*ΔTM9SF4*^+/-^ cell line was serially diluted by a factor of 10 until 0.5–1 cell per well was seeded into a 96-well plate. One day after cell attachment, observed and documented single-cell Wells under an optical microscope (Olympus, Japan). The cells were then cultured for a week. The single-cell populations were gradually expanded from the 96-well plate to a 24-well plate, then to a 6-well plate, and finally to a T25 cell culture flask.

After being correctly identified by sequencing, the cells were passaged and cryopreserved. The identification primers were based on the *TM9SF4* deletion identification primers listed in **Table 1**. The expression of TM9SF4 was verified by western blotting and quantitative real-time PCR. In the following sections of this paper, this cell line was designated as PK15-*TM9SF4*-KO.

### 2.11 Construction of *TM9SF4* overexpressing cells

Based on the coding DNA sequence (CDs) of *TM9SF4* (XM_005672825.3) provided by NCBI, an overexpression plasmid was designed and constructed using the pCDH-CMV-MCS-EF1-copGFP-T2A-Puro vector. Subsequently, the recombinant plasmid pCDH-CMV-MCS-EF1-copGFP-T2A-Puro-*TM9SF4* was co-transfected with psPAX and pMD2.G into HEK-293T cells using Lipofectamine 3000, and the supernatant containing lentivirus was collected 48 hours later. The lentiviral supernatant was filtered through a 0.22μM filter and infected PK15 cells for 48 hours. Then, the cells were cultured in fresh medium containing 5 μg/mL puromycin (Beyotime, China) to select positive cells. The expression of TM9SF4 was verified by western blotting and quantitative real-time PCR. Thus, the PK15-*TM9SF4*-OE stable cell line was obtained.

### 2.12 Cell viability assay

The cytotoxicity induced by *Gp*CDT was assessed using a Cell Counting Kit-8 (CCK-8) (Coolaber, China). According to the manufacturer’s instructions, PK15, PK15*ΔEPHB4*^+/-^, PK15*ΔLITAF*^+/-^, PK15*ΔTM9SF4*^+/-^, PK15*ΔSKLC12A4*^+/-^, PK15-*TM9SF4*-KO, and PK15-*TM9SF4*-OE cell lines were seeded into 96-well plates at a density of 1×10^4^ cells per well and treated with *Gp*CDT at concentrations of 0 μg/mL, 1 μg/mL, 10 μg/mL, and 100 μg/mL. After 48 hours, add 10 μL of CCK-8 solution and incubate for 1 hour at 37°C. The absorbance was measured at 450 nm using a microplate reader (Bio-Rad, USA). Each sample underwent three biological replicates. Additionally, PK15 cells exhibited the most significant damage following a 48-hour exposure to 10 μg/mL *Gp*CDT. Therefore, cytopathic effects were examined under an optical microscope, and representative images were captured (Olympus, Japan) (Xu et al., 2025).

### 2.13 Western blotting

Cell lysates were prepared using either Nondenaturing Lysis Buffer (Solarbio, China) supplemented with 1 mM PMSF or RIPA Lysis Buffer (Coolaber, China). The lysates were subsequently mixed with 5× protein loading buffer and denatured by heating in a boiling water bath for 5 minutes or at 37 °C for 30 minutes (applied for SEC63, TM9SF4 and SLC12A4 proteins to prevent aggregation due to high temperature). For Co-IP samples, the beads were washed seven times with cold PBST and then resuspended in Nondenaturing Lysis Buffer containing 5× protein loading buffer, followed by boiling for 5 minutes.

Proteins were separated by Omni-Easy™ One-step Color PAGE Gel Rapid Preparation Kit (Epizyme Biotech, China) and transferred onto PVDF membranes (Vazyme, China). The membranes were blocked with 5% milk at room temperature for 2 hours, then incubated with rabbit anti-Myc-tag pAb (1:5000, HUABIO, China), mouse anti-His-tag mAb (1:5000, ABclonal, China), rabbit anti-β-actin mAb (1:80000, ABclonal, China), rabbit anti-GAPDH pAb (1:5000, Proteintech, China) overnight at 4 °C, and rabbit anti-TM9SF4 pAb (1:4000, Proteintech, China) for 1 hour at 37 °C. After incubation, the membranes were washed five times with TBST and then incubated with HRP-conjugated goat anti-rabbit or goat anti-mouse IgG antibody (1:5000, BOSTER, USA) at room temperature for 1 hour, followed by three washes with TBST. Protein signals were detected using an enhanced chemiluminescence (ECL) reagent (Bio-Rad, USA). The protein molecular weight was determined using the ColorMixed Protein Marker (10–180 kDa, ABclonal, China).

### 2.14 RNA extraction and quantitative real-time PCR

The PK15, PK15-*TM9SF4*-KO, and PK15-*TM9SF4*-OE cells were collected from T25 flasks. Total RNA was extracted using the SteadyPure Quick RNA Extraction Kit (Accurate Biology, China). cDNA synthesis was performed with HiScript II RT SuperMix for qPCR (Vazyme, China), followed by the design of specific primers for *TM9SF4* (**Table 1**). The mRNA expression levels were normalized to β-actin and GAPDH. Quantitative real-time PCR of the transcripts was conducted using Taq Pro Universal SYBR qPCR Master Mix (Vazyme, China). The qPCR cycling conditions consisted of an initial denaturation at 95 °C for 30 seconds, followed by 40 cycles of amplification: 95 °C for 10 seconds and 62 °C for 30 seconds. Following the final amplification cycle, a melt curve analysis was performed (65 °C to 95 °C) to verify amplification specificity. Each sample underwent three biological replicates.

### 2.15 Indirect immunofluorescence assay

The PK15, PK15-*TM9SF4*-KO and PK15-*TM9SF4*-OE cells were seeded at a density of 5 × 10^5^ in 6-well plates containing climbing film. Subsequently, the cells were exposed to *Gp*CDT at a concentration of 10 μg/mL or stained with a red membrane probe Dil (Beyotime, China) at a concentration of 5 μM. Incubate at 4 °C for 30 minutes to ensure *Gp*CDT or Dil binds tightly to the cell membrane. Afterwards, the cells were washed three times with cold PBS and fixed in 4% paraformaldehyde at 4 °C for 30 minutes. The cells were again washed three times with PBS and blocked using 5% bovine serum albumin (BSA) (Coolaber, China) for one hour at room temperature. The cells were then incubated overnight at 4 °C with mouse anti-His-tag mAb (1:200) and rabbit anti-TM9SF4 pAb (1:200). After washing with PBST, fluorescently labeled secondary antibodies DyLight 488 Conjugated AffiniPure Goat Anti-Rabbit IgG (H+L) (1:200) (BOSTER, USA) and DyLight 594 Conjugated AffiniPure Goat Anti-Mouse IgG (H+L) (1:200) (BOSTER, USA) were added and incubated for one hour at room temperature. Following another round of washes, the climbing film containing the samples were covered on slides using a Antifade Mounting Medium with DAPI (Beyotime, China). Finally, the cells were observed using an upright fluorescence microscope (Olympus BX53F2C, Japan), and the most representative images were captured. All images were collected and displayed at the same exposure time: TM9SF4 membrane protein channel 3.668s; *Gp*CDT toxin channel 4s; the Dil cell membrane channel is 960.2ms; DAPI channel 201.3ms. Three biological replicates were conducted, and the immunofluorescence signals were quantitatively analyzed using ImageJ software.

### 2.16 Statistical analysis

Experimental data were statistically analyzed using GraphPad Prism version 8.0. An unpaired Student’s t-test and one-way analysis of variance (ANOVA) were employed for comparisons between groups. Statistical significance was defined as follows: *p < 0.05, **p < 0.01 and ***p < 0.001. Data were expressed as mean ± standard deviation (SD), all the experiments were repeated at least three times.

## 3 Results

### 3.1 Identification of 9 host cell proteins that may interact with *Gp*CDT

**Figure 1:**
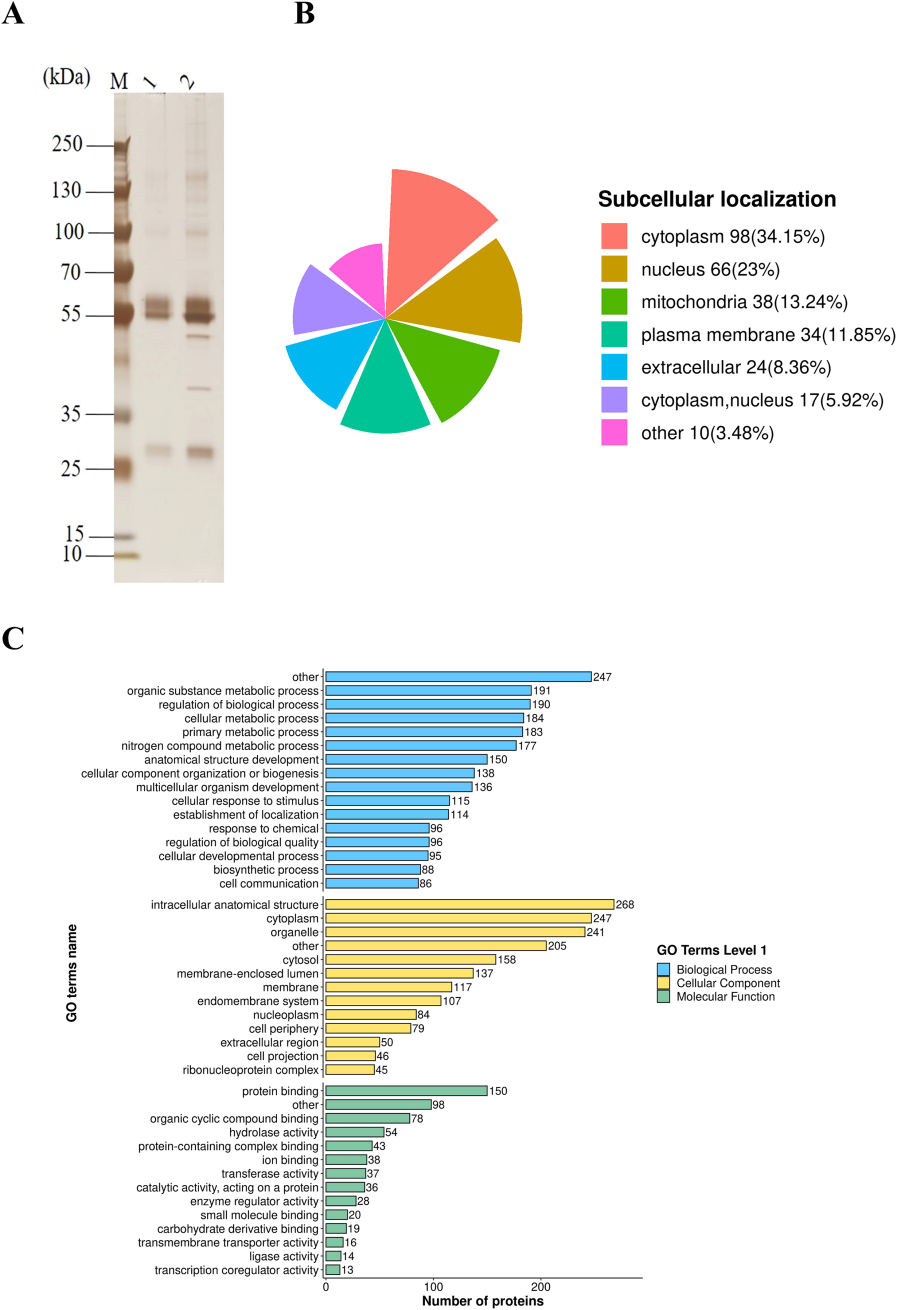
LC-MS/MS analysis initially identified a series of host cell proteins potentially interacting with *Gp*CDT. **(A)** Co-IP of *Gp*CDT and PK15 membrane proteins. Lane 1, antibody-linked beads, His-tagged protein and membrane proteins; Lane 2, antibody-linked beads, *Gp*CDT and membrane proteins. **(B)** Subcellular localization of upregulated proteins. **(C)** Gene Ontology classification of upregulated proteins.

The purified recombinant *Gp*CDT protein was separated and validated by 10% SDS-PAGE, and the purified His-tagged control protein was confirmed by Western blotting analysis (**Supplementary Figures 1A, B**). These preparations provided essential biological materials for subsequent experiments. In **Figures 1A**, compared, to the control (lane 1), the immunoprecipitation with *Gp*CDT (lane 2) displays several distinct bands of varying intensity, suggesting the presence of multiple host cell proteins that may specifically bind to *Gp*CDT. Consequently, LC-MS/MS analysis of the bead samples identified 287 significantly upregulated proteins in the *Gp*CDT immunoprecipitation group (**Supplementary Figure 1C**). **Figure 1B** shows the subcellular localization of these upregulated proteins, with 58 proteins localized to the plasma membrane and extracellular space. Based on GO classification (**Figure 1C**), from the above 58 proteins, we selected nine candidate proteins involved in signal transduction, transmembrane transport, receptor complex, signaling receptor activity and cell adhesion (**Table 2**) for further investigation as potential *Gp*CDT receptors.

**Table 2:** Host cell proteins that may interact with *Gp*CDT.

Number	Protein accession	Gene names	Subcellular localization	Molecular function
1	A0A287AYF2	EPHB4	Extracellular	receptor complex
2	A0A5G2QK84	ITGA5	Plasma membrane	receptor complex
3	A0A286ZTE6	APP	Extracellular	receptor complex
4	I3LPG3	UGCG	Extracellular	signal transduction
5	A0A4X1U746	GOLGA7	Extracellular	transmembrane transport
6	A0A4X1VJC1	LITAF	Plasma membrane	transmembrane transport
7	A0A287BSC9	TM9SF4	Plasma membrane	cell adhesion
8	A0A287BAA6	SLC12A4	Plasma membrane	transmembrane transport
9	A0A286ZNJ7	SEC63	Plasma membrane	signaling receptor activity

### 3.2 Verify the interaction between host cell proteins and *Gp*CDT

The successfully constructed recombinant plasmids—pcDNA3.1-Myc-EPHB4, pcDNA3.1-Myc-ITGA5, pcDNA3.1-Myc-APP, pcDNA3.1-Myc-UGCG, pcDNA3.1-Myc-GOLGA7, pcDNA3.1-Myc-LITAF, pcDNA3.1-Myc-TM9SF4, pcDNA3.1-Myc-SLC12A4, and pcDNA3.1-Myc-SEC63—along with the pcDNA3.1-Myc empty vector, were individually transfected into HEK-293T cells for 48 hours. Expression of all nine Myc-tagged proteins in cell lysates was confirmed using a rabbit anti-Myc-tag pAb (**Supplementary Figure 2**). To assess whether these host proteins interact with *Gp*CDT, co-immunoprecipitation (IP) and Western blotting (IB) assays were performed using two different tag antibodies: either an anti-Myc tag antibody or an anti-His tag antibody, targeting total cell lysates or *Gp*CDT, respectively.

**Figure 2:**
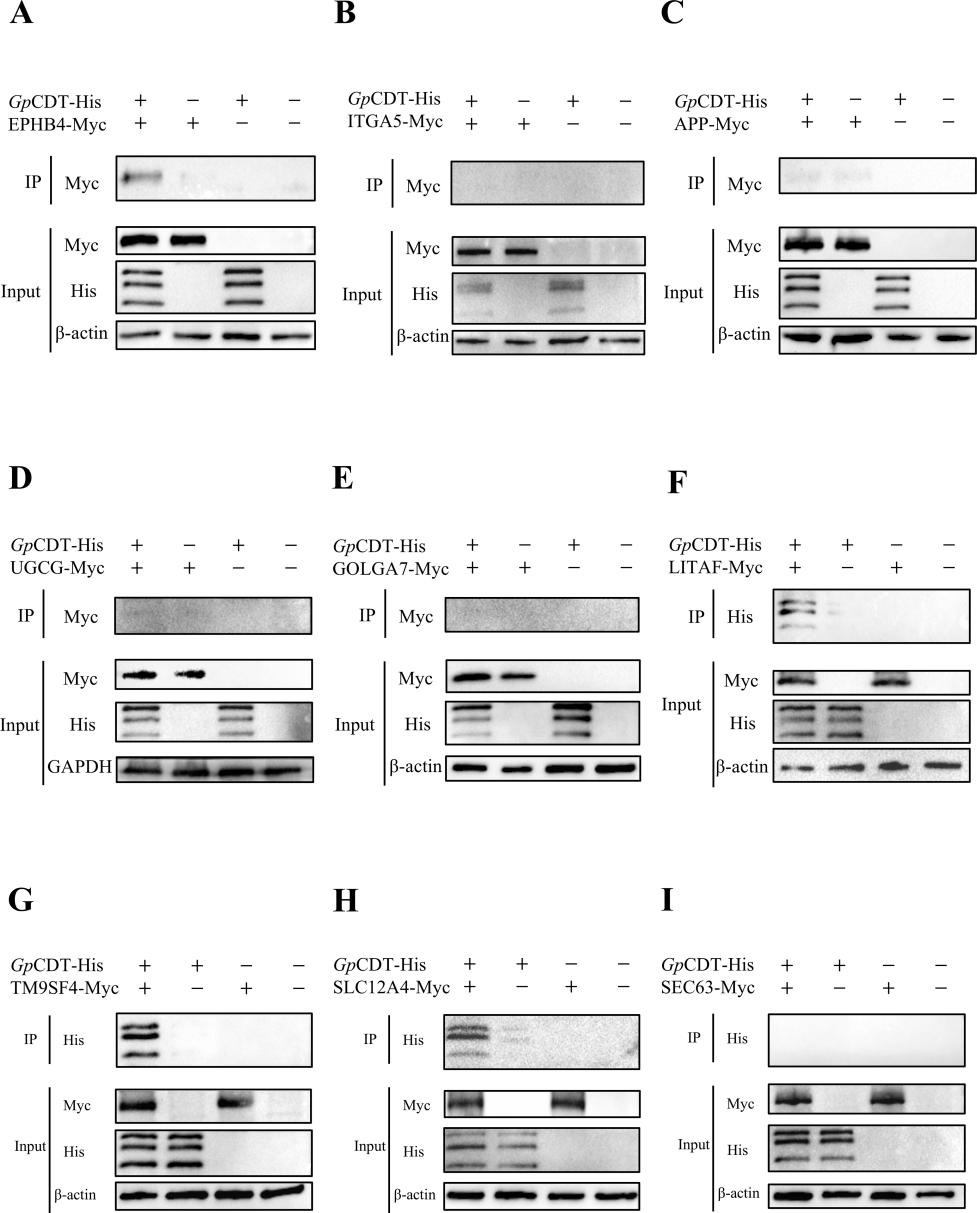
Validate the interaction between host proteins and *Gp*CDT based on Co-IP. **(A)** The *Gp*CDT-His protein (2μg) was immunoprecipitated with anti-His-tag mAb, and the EPHB4 or **(B)** ITGA5, **(C)** APP, **(D)** UGCG, **(E)** GOLGA7 was specifically detected with anti-Myc-tag pAb. **(F)** The LITAF or **(G)** TM9SF4, **(H)** SLC12A4, **(I)** SEC63 whole-cell lysates (200μL) was immunoprecipitated with anti-Myc-tag pAb, and the *Gp*CDT-His was specifically detected with anti-His-tag mAb.

As shown in **Figures 2A**, a specific band was detected in samples immunoprecipitated with anti-*Gp*CDT-His antibody (**Figures 2A**, lane 1), which corresponded to the apparent molecular weight of EPHB4-Myc (125 kDa). No band was observed in the negative control using His-tagged protein (**Figures 2A**, lane 2). The presence of EPHB4-Myc in the cell lysates was verified in the Input group, ruling out non-specific binding. Furthermore, *Gp*CDT-His was specifically detected in samples immunoprecipitated with anti-LITAF-Myc, anti-TM9SF4-Myc, and anti-SLC12A4-Myc antibodies (**Figures 2F–H**, lane 1), with band sizes consistent with the purified *Gp*CDT shown in **Supplementary Figure 1A**. No bands were observed in the Myc-tagged protein negative controls (**Figures 2F–H**, lane 2). Thus, these results demonstrate that EPHB4, LITAF, TM9SF4, and SLC12A4 specifically interact with *Gp*CDT.

### 3.3 Effect of *EPHB4*, *LITAF*, *TM9SF4* and *SLC12A4* gene polyclonal knockout on the *Gp*CDT virulence

**Figure 3:**
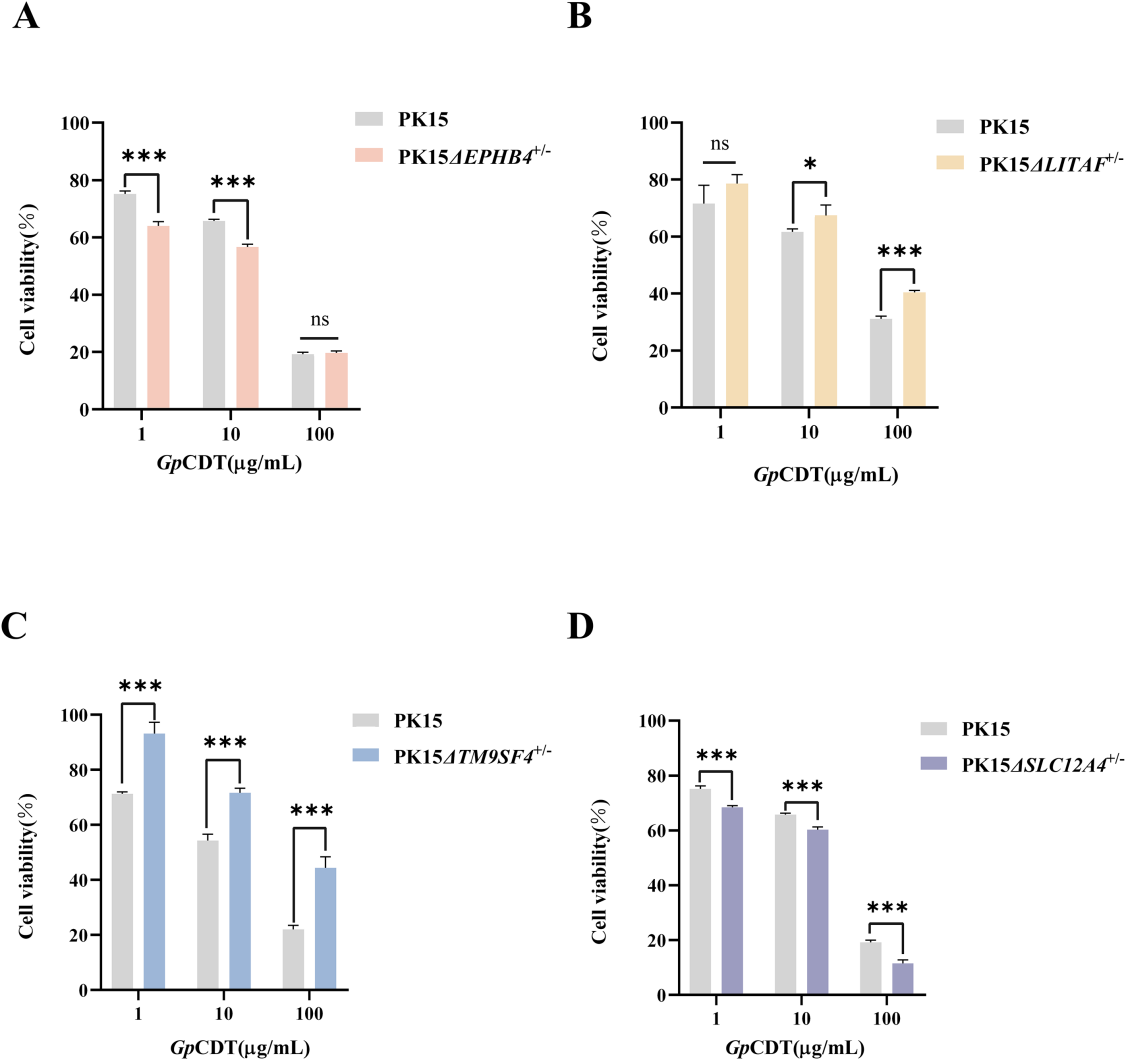
The viability of polyclonal knockout PK15 cell lines after exposure to *Gp*CDT. **(A-D)***Gp*CDT was inoculated into PK15 and polyclonal knockout PK15 cell lines with 1 μg/mL, 10 μg/mL, 100 μg/mL for 48h, the viability as determined by CCK-8 reagent at 450 nm. An unpaired Student’s t-test was employed for comparisons between groups. Data are shown as mean ± SD (n = 4). (ns, *p*> 0.05; *p < 0.05; ***p < 0.001).

Having established that EPHB4, LITAF, TM9SF4, and SLC12A4 can interact with *Gp*CDT, we next investigated their functional roles in mediating the toxin’s virulence. Using CRISPR-Cas9, we successfully generated PK15 polyclonal knockout cell lines for each gene: PK15*ΔEPHB4*^+/-^, PK15*ΔLITAF*^+/-^, PK15*ΔTM9SF4*^+/-^, and PK15*ΔSLC12A4*^+/-^, and the CCK-8 assay showed that knockout of these genes did not significantly affect baseline cell viability (**Supplementary Figure 3**). Subsequently, *Gp*CDT was inoculated into PK15 cells and polyclonal knockout cell lines at concentrations of 1 μg/mL, 10 μg/mL, and 100 μg/mL. The most pronounced resistance to *Gp*CDT virulence was observed in PK15*ΔTM9SF4*^+/-^ cells, which exhibited increases in viability of 21.93%, 17.39%, and 22.43% compared to PK15 cells at the respective concentrations (**Figure 3C**), indicating that *TM9SF4* knockout attenuates *Gp*CDT toxicity. In contrast, *LITAF* knockout provided only a minor protection (7.02%, 5.82%, and 9.21% inhibition) (**Figure 3B**). In addition, polyclonal knockout of the *EPHB4* and *SLC12A4* genes resulted in a reduced cell survival rate (**Figures 3A, D**).

### 3.4 TM9SF4 acts as a host cell receptor for the internalization of *Gp*CDT toxin to induce cytotoxicity

Based on the above results, which indicated that TM9SF4 plays an important role in the process of *Gp*CDT exerting cytotoxicity on host cells, we proceeded to generate a homozygous PK15-*TM9SF4*-KO clone line (KO) via limited dilution and constructed a stable overexpression line of PK15-*TM9SF4*-OE (OE) to further investigate the role of TM9SF4 in *Gp*CDT cytotoxicity. Sequencing analysis of KO cells, along with the mRNA and protein expression levels of TM9SF4 in both KO and OE cells, collectively confirmed the successful establishment of the PK15-*TM9SF4*-KO and PK15-*TM9SF4*-OE cell lines (**Supplementary Figures 4A–C**). The CCK-8 assay revealed that neither knockout nor overexpression of *TM9SF4* significantly affected baseline cell viability (**Supplementary Figure 4D**).

**Figure 4:**
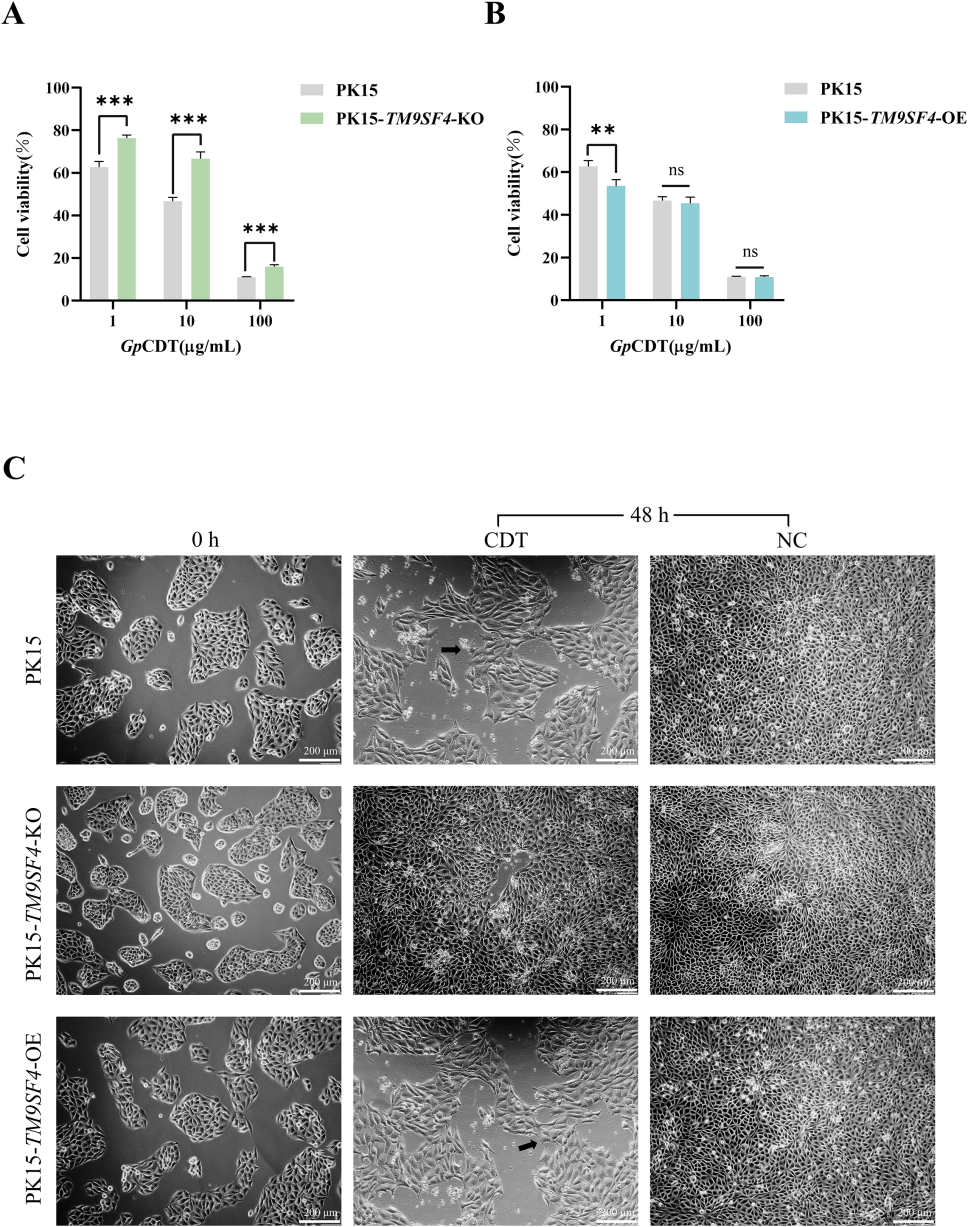
Cytotoxicity of *Gp*CDT in PK15, PK15-*TM9SF4*-KO, and PK15-*TM9SF4*-OE cells. **(A-B)***Gp*CDT was inoculated into PK15, PK15-*TM9SF4*-KO and PK15-*TM9SF4*-OE cell lines with 1 μg/mL, 10 μg/mL, 100 μg/mL for 48h, the viability as determined by CCK-8 reagent at 450 nm. Data are shown as mean ± SD (n = 4). Statistical significance was determined by an unpaired Student’s t-test. (ns, *p* > 0.05; **p < 0.01; ***p < 0.001). **(C)** The PK15, PK15-*TM9SF4*-KO and PK15-*TM9SF4*-OE cells were seeded into 6-well plates. Once the cell confluence reaches 60 - 70%, challenge the cells with *Gp*CDT at a concentration of 10 μg/mL. After 48 hours, observe the cytopathic effects and capture the most representative images (magnification: 100 ×; scale bar: 200 µm). (KO: knockout; OE: overexpression; NC: negative control).

We next evaluated the impact of TM9SF4 on cellular susceptibility to *Gp*CDT. We challenged these cell lines with *Gp*CDT at 1 μg/mL, 10 μg/mL and 100 μg/mL for 48 hours. The viability of KO cells was significantly increased by 13.61%, 20.03%, and 4.97%, respectively, compared to PK15 cells (**Figure 4A**). Conversely, OE cells exhibited a significant reduction in viability (9.28%) at the 1 μg/mL concentration, but showed no significant difference from PK15 cells at higher concentrations (**Figure 4B**). Furthermore, to examine the effect of TM9SF4 on *Gp*CDT-induced Cytopathic Effect (CPE), we observed the cells under an optical microscope after 48 hours of exposure to 10 μg/mL *Gp*CDT. PK15 and OE cells displayed typical CPE—including cellular distention, pleomorphism, blurred boundaries, and substantial cell death, the KO population remained largely unaffected, exhibiting a normal morphology and nearly confluent growth (**Figure 4C**). This result demonstrates that the absence of TM9SF4 markedly protects cells from *Gp*CDT-induced cytopathology.

**Figure 5:**
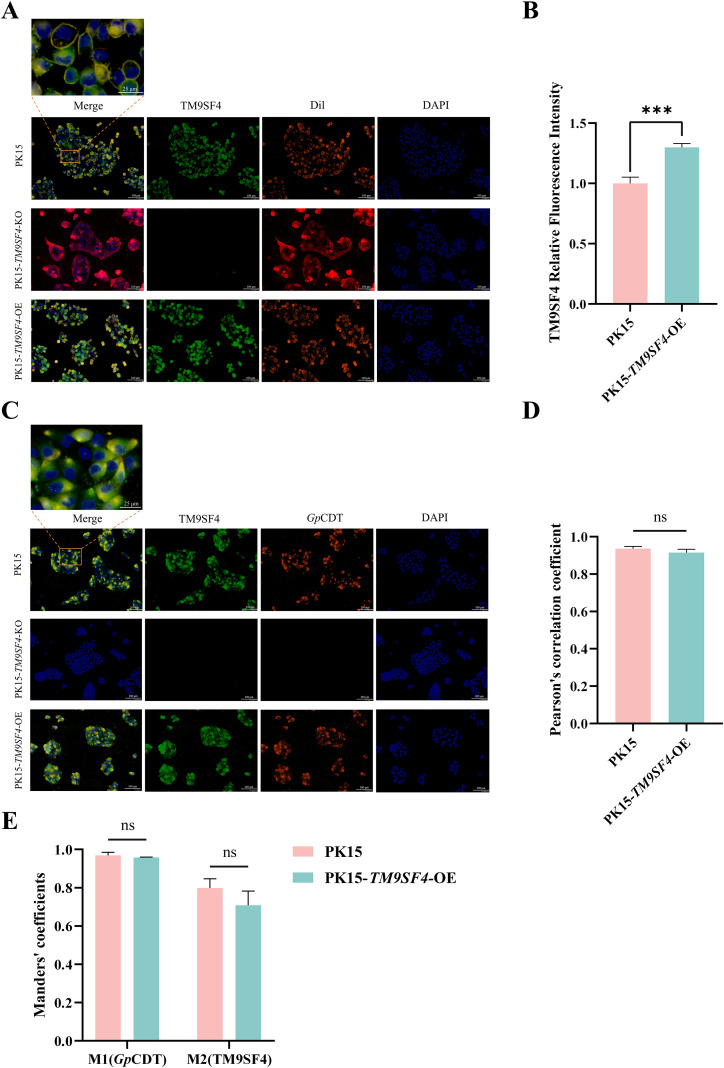
TM9SF4 host protein colocalization with plasma membrane and *Gp*CDT. **(A)** IFA was used to detect the co-localization of TM9SF4 protein with the plasma membrane. TM9SF4 (green fluorescence), Dil (red fluorescent probe of the cell membrane), and DAPI (blue fluorescent dye of the cell nucleus) (magnification: 200 ×; scale bar: 100µm, 25 µm). **(B)** The relative fluorescence intensity of TM9SF4 in PK15 and PK15-*TM9SF4*-OE cells was quantified using ImageJ. Statistical significance was determined by an unpaired Student’s t-test. Data are shown as mean ± SD (n = 3), ***p < 0.001. **(C)** IFA was used to detect the co-localization of TM9SF4 and *Gp*CDT in plasma membranes and endocytic vesicles. TM9SF4 (green fluorescence), *Gp*CDT (red fluorescence) and DAPI (blue fluorescent dye of the cell nucleus) (magnification: 200 ×; scale bar: 100µm, 25 µm). **(D, E)** The co-localization of TM9SF4 and *Gp*CDT in PK15 and PK15-*TM9SF4*-OE cells was quantitatively analyzed using ImageJ software with JACoP plugin. The data are presented using Pearson’s correlation coefficient and Manders’ overlap coefficients. Here, M1 represents the proportion of *Gp*CDT toxins overlapping with the TM9SF4 membrane protein, and M2 represents the proportion of TM9SF4 membrane proteins overlapping with the *Gp*CDT toxin. Statistical significance was determined by an unpaired Student’s t-test. Data are shown as mean ± SD (n = 3), ns, *p* > 0.05. (KO: knockout; OE: overexpression. All images were collected and displayed at the same exposure time: TM9SF4 membrane protein channel 3.668s; *Gp*CDT toxin channel 4s; The Dil cell membrane channel is 960.2ms; DAPI channel 201.3ms).

To further validate the interaction between TM9SF4 and *Gp*CDT, we performed an indirect immunofluorescence assay to examine their co-localization. We first confirmed the plasma membrane localization of TM9SF4 by co-staining with the lipophilic dye Dil. In both PK15 and OE cells, TM9SF4 (green) showed clear co-localization (yellow) with the plasma membrane (red), confirming TM9SF4 as a membrane protein. As expected, no specific TM9SF4 signal was detected in KO cells under identical exposure settings (**Figure 5A**). Quantitative analysis of the fluorescence intensity further confirmed successful TM9SF4 overexpression in the OE line (**Figure 5B**). We next incubated the cells with 10 μg/mL *Gp*CDT at 4 °C for 30 minutes to ensure complete binding of the toxins to the plasma membrane. Strikingly, in both PK15 and OE cells, TM9SF4 (green) and *Gp*CDT (red) exhibited extensive co-localization (yellow) at the plasma membrane and in endocytic vesicles. In contrast, the KO cells showed negligible binding of *Gp*CDT, with signals reduced to background levels (**Figure 5C**). Quantitative co-localization analysis in PK15 cells revealed that the Pearson’s correlation coefficient (PCC) was 0.936, >0.5, indicating a strong correlation (**Figure 5D**). Furthermore, Mander’s coefficient M1 was 0.967, which is close to 1, demonstrating that nearly all internalized toxin was associated with TM9SF4 (**Figure 5E**). These quantitative metrics could not be calculated for the KO cells due to the absence of signal above background. The OE line showed no significant difference in these coefficients compared to PK15 cells. Collectively, these results demonstrate that TM9SF4 severs as a host cell receptor essential for the binding, internalization, and cytotoxicity of *Gp*CDT.

## 4 Discussion

*Glaesserella parasuis* colonizes the upper respiratory tract of healthy pigs without causing disease. However, under conditions of stress or co-infection with other pathogens, it can lead to systemic Glässer’s disease, which is characterized by fibrinous polyserositis, polyarthritis, and meningitis (Samantha J et al., 2021; Wang et al., 2021). The *Gp*CDT toxin is a major virulence factor of *G. parasuis* and plays a critical role in its pathogenesis. The binding of a toxin to a host cell receptor is the initial step in its intoxication process. Previous studies have indicated that N-linked glycoproteins, glycosphingolipids, TMEM181, SYNGR2, TMEM127, and GPR107 may serve as receptors for *Ec*CDT, *Aa*CDT, *Hd*CDT, and *Cj*CDT (Du and Song, 2022). However, the identity of the host cell receptor for *Gp*CDT remains unknown.

To identify the host cell receptor of *Gp*CDT, we screened for interacting host proteins in PK15 cells using co-immunoprecipitation combined with LC-MS/MS (Co-IP/LC-MS/MS). This approach identified 287 significantly enriched proteins. Based on Subcellular localization analysis, a total of 58 proteins localized to the plasma membrane and extracellular domains were identified. By integrating Gene Ontology (GO) functional classification and previously published data, we selected nine putative receptors for further validation: EPHB4, ITGA5, APP, UGCG, GOLGA7, LITAF, TM9SF4, SLC12A4, and SEC63. Subsequent Co-IP experiments confirmed that four of these—EPHB4, LITAF, SLC12A4, and TM9SF4—specifically interact with *Gp*CDT. EPHB4 is a receptor tyrosine kinase (RTK). Although no direct association with bacterial toxins has been reported, it is a recognized therapeutic target in oncology for blocking pro-oncogenic signaling (Chen et al., 2019). LITAF was identified as the host receptor for *Bacillus cereus* hemolysin BL (HBL), cells and mice deficient in LITAF show significant resistance to HBL-induced lethality, highlighting its potential as a target for antitoxin therapy (Liu et al., 2020; Enosi Tuipulotu et al., 2021). SLC12A4 is a serine transporter that regulates immune responses by modulating the phagocytosis of apoptotic cells by macrophages, which may indirectly influence the host’s capacity to clear bacterial toxins (Perry et al., 2019). TM9SF4, a multi-pass transmembrane protein belonging to the Transmembrane 9 Superfamily, has been shown to be required for the adhesion and internalization of pathogenic Gram-negative bacteria in *Drosophila* cells. TM9SF4 loss significantly reduces host susceptibility to these bacteria (Bergeret et al., 2008). Furthermore, a haploid genetic screen identified TM9SF4 as a host factor closely associated with the binding of *Cj*CDT to host cells (Carette et al., 2011), suggesting its potential role as a receptor for CDTs.

In this study, when using eukaryotic expression of HEK-293T cells and Western bloting verification, the apparent molecular weights of EPHB4-Myc, ITGA5-Myc, APP-Myc and TM9SF4-Myc proteins do not match the theoretical molecular weights of 112kDa, 119kDa, 90kDa and 60kDa. The observed higher molecular weights may be due to post-translational modifications of the membrane proteins, such as glycosylation (Isaji et al., 2006; Lin et al., 2022; Lyu et al., 2025). Moreover, TM9SF4-Myc proteins may form oligomers or higher-order assemblies, potentially contributing to slower migration.

Cell viability served as a direct metric to assess the impact of gene knockout on *Gp*CDT toxicity. Among the generated heterozygous knockout lines (PK15*ΔEPHB4*^+/-^, PK15*ΔLITAF*^+/-^, PK15*ΔSLC12A4*^+/-^, and PK15*ΔTM9SF4*^+/-^), only PK15*ΔTM9SF4*^+/-^ exhibited significant resistance to *Gp*CDT across all tested concentrations (1, 10, and 100 μg/mL), establishing *TM9SF4* as our primary research focus. In contrast, knockout of *EPHB4* or *SLC12A4* paradoxically increased cellular susceptibility to the toxin. We hypothesize that these proteins do not act as pro-infection receptors but may instead contribute to toxin clearance or cellular repair under stress conditions. For instance, EPHB4 interacts with the insulin receptor (InsR) and directs its lysosomal degradation (Liu et al., 2022). *Gp*CDT by binding to EPHB4, might exploit this endocytic machinery for entry. However, *EPHB4* knockout could disrupt this protective degradation pathway, inadvertently impairing toxin clearance and exacerbating poisoning. Similarly, SLC12A4, a solute carrier, promotes phagocytic activity in immune cells. For example, the gut commensal metabolite rhamnose binds to SLC12A4, activating the small GTPases Rac1 and Cdc42, thereby promoting macrophage phagocytosis (Li et al., 2024). The absence of *SLC12A4* may therefore impair clearance of *Gp*CDT, resulting in reduced cell viability.

Validation of TM9SF4’s role as a receptor revealed a concentration-dependent phenotype. Notably, *TM9SF4* overexpression significantly enhanced cellular susceptibility to a low toxin dose (1 μg/mL), while this effect was saturated at higher concentrations (10, 100 μg/mL). Consistent with this concentration-dependent effect, quantitative co-localization analysis (IFA) at 10 μg/mL *Gp*CDT revealed no significant difference in Pearson’s or Mander’s coefficients between PK15-*TM9SF4*-OE and PK15 cells, aligning with the CCK-8 results. We speculate that at low toxin concentrations, overexpression of *TM9SF4* may increase the number of available cell surface receptors and enhance trafficking efficiency, thereby promoting toxin internalization. However, when toxin levels are high, the endocytic and sorting machinery—including clathrin, adaptor proteins, V-ATPase-associated pathways, and early/late endosomal processing—may become saturated or diverted to alternative, non-productive routes. These alternative pathways may involve a shift from efficient receptor-mediated endocytosis to less effective or degradative uptake mechanisms. Such saturation or switching of trafficking pathways can result in a bell-shaped or biphasic dose–response curve, characterized by enhanced uptake at low doses but reduced or unchanged infection at high doses. Similar non-linear dynamics have been observed in other receptor–ligand systems, such as EGFR, where receptor overexpression or variations in ligand concentration influence receptor ubiquitination, signaling kinetics, and endocytic trafficking (Capuani et al., 2015). Moreover, studies indicate that TM9SF4 interacts with V-ATPase and modulates endosomal acidification, suggesting that its overexpression may affect toxin processing through alterations in vesicular trafficking and pH homeostasis (Lozupone et al., 2015).

In this study, we demonstrated that the absence of TM9SF4 confers significant resistance to *Gp*CDT intoxication and enhances cell survival. In line with its established role as a DNase I-type toxin, CDT is widely reported to cause DNA damage and induce p53-dependent apoptosis following cellular internalization (Mao et al., 2023). It has also been shown to trigger pyroptosis in a dose- and time-dependent manner via the ROS/caspase-9/caspase-3/GSDME pathway (Gu et al., 2022), and to induce ferroptosis by disrupting redox homeostasis (Xu et al., 2025). An unresolved question from our work is how TM9SF4 influences the specific mode of cell death activated by *Gp*CDT. To address this, future studies will systematically assess changes in key marker across different cell death pathways, including apoptosis (cleaved Caspase-3), pyroptosis (GSDMD, GSDME), ferroptosis (GSH, MDA, ROS), and previously unexamined pathways for *Gp*CDT such as cuproptosis (FDX1, LIAS) and autophagic cell death (LC3-II) (Eskander et al., 2025; Mao et al., 2025).

It is well-established that CDT is a AB2 type heterotrimeric holotoxin formed by CdtA, CdtB and CdtC subunits (Jinadasa et al., 2011). CdtB serves as the biological active subunit, possessing both DNase I and phosphatidylinositol-3,4,5-trisphosphate (PIP_3_) phosphatase activities (Shenker et al., 2007; Chen et al., 2017). In contrast, CdtA and CdtC subunit lack catalytic activity and function as binding subunits that mediate attachment to specific receptors on target cells (Frisan, 2016). The binding mechanism of these subunits appears complex; some studies suggest that CdtA and CdtC can bind to cells independently and may compete for receptor interaction (Lee et al., 2003; McSweeney and Dreyfus, 2004; Cao et al., 2008), while others propose that they function as a heterodimer to cooperatively facilitate holotoxin binding (Deng and Hansen, 2003; DiRienzo, 2014). However, our study has thus far only confirmed the interaction between the *Gp*CDT holotoxin and the host cell receptor TM9SF4 in PK15. Several questions remain unresolved: namely, whether individual subunits (*Gp*CdtA, *Gp*CdtB, *Gp*CdtC) can bind TM9SF4 independently, and which specific domain(s) of TM9SF4 are responsible for mediating this interaction.

While this study initially establishes that TM9SF4 functions as a receptor and play an important role in mediating *Gp*CDT toxin–induced cytotoxicity in PK15 cells. However, whether it is the sole receptor for *Gp*CDT on PK15 cells requires further validation. Furthermore, it remains unclear whether this protein serves a similar receptor function in other pertinent porcine host cells, such as newborn pig tracheal epithelial (NPTr) cells or porcine alveolar macrophages (3D4/21). Therefore, we will systematically investigate the interaction between *Gp*CDT and TM9SF4 across these different host cell types. This work aims to provide comprehensive evidence to establish TM9SF4 as a universal and critical receptor for *Gp*CDT.

In summary, we identified nine candidate host cell receptors for *Gp*CDT via Co-IP and LC-MS/MS. Among them, TM9SF4 was characterized as a receptor for *Gp*CDT on PK15 cells, based on evidence of its direct interaction with the purified *Gp*CDT toxin, its important role in mediating toxin-induced cytotoxicity (assessed by cell viability and CPE assays), and its strong co-localization with *Gp*CDT, confirmed by IFA. Identifying the cellular receptor for *Gp*CDT is pivotal for comprehensively elucidating its cytotoxic mechanism. This finding not only provides a foundation for a deeper understanding of *G. parasuis* pathogenesis but also opens new avenues for developing targeted strategies against swine Glässer’s disease.

## Data Availability

The datasets presented in this study can be found in online repositories. The names of the repository/repositories and accession number(s) can be found in the article/**Supplementary Material**.

## Supplementary material

Supplementary Figure 1 Preparation of biological materials for LC-MS/MS analysis and identification of differentially expressed proteins. **(A)** SDS–PAGE analysis of purified *Gp*CDT. **(B)** Western blotting of purified His-tagged protein. **(C)** Differentially regulated proteins identified by LC-MS/MS.

Supplementary Figure 2 Western blotting of 9 host cell proteins expression in HEK-293T Cells. **(A-I)** pcDNA3.1-Myc-EPHB4, pcDNA3.1-Myc-ITGA5, pcDNA3.1-Myc-APP, pcDNA3.1-Myc-UGCG, pcDNA3.1-Myc-GOLGA7, pcDNA3.1-Myc-LITAF, pcDNA3.1-Myc-TM9SF4, pcDNA3.1-Myc-SLC12A4, pcDNA3.1-Myc-SEC63 and pcDNA3.1-Myc Vector were transfected into HEK-293T for 48h. The eukaryotic expression of the protein was detected using rabbit anti-Myc-tag pAb.

Supplementary Figure 3 Construction and validation of PK15 polyclonal gene knockout cell lines. **(A)** DNA sequencing analysis of *EPHB4*, *LITAF*, *TM9SF4* and *SLC12A4* polyclonal knockout PK15 cell lines. **(B)** The PK15 cell and polyclonal knockout PK15 cell lines were seeded into 96-well microplates and cultured for 48 h. Cell viability as determined by CCK-8 reagent at 450 nm. One-way analysis of variance (ANOVA) was employed for comparisons between groups. Data are shown as mean ± SD (n = 4), ns, *p* > 0.05.

Supplementary Figure 4 Construction and validation of PK15-*TM9SF4*-KO and PK15-*TM9SF4*-OE. **(A)** Sequencing alignment analysis of *TM9SF4* gene in PK15 and PK15-*TM9SF4*-KO. **(B)** Western blotting the TM9SF4 protein level in PK15, PK15-*TM9SF4*-KO and PK15-*TM9SF4*-OE. TM9SF4 levels were quantified by ImageJ. **(C)** qRT-PCR the relative *TM9SF4* mRNA level in PK15, PK15-*TM9SF4*-KO and PK15-*TM9SF4*-OE. An unpaired Student’s t-test was employed for comparisons between groups. Data are shown as mean ± SD (n = 3), ****p* < 0.001. **(D)** The PK15, PK15-*TM9SF4*-KO and PK15-*TM9SF4*-OE cell lines were seeded into 96-well plates and cultured for 48 h. Cell viability as determined by CCK-8 reagent at 450 nm. One-way analysis of variance (ANOVA) was employed for comparisons between groups. Data are shown as mean ± SD (n = 4), ns, *p* > 0.05. (KO: knockout; OE: overexpression).
