# Supplementary figures and images for: TM9SF4 acts as a receptor mediating Glaesserella parasuis cytolethal distending toxin–induced cytotoxicity in PK15 cells
Source: Front Cell Infect Microbiol. 2026 Mar 19;16:1783709. doi: 10.3389/fcimb.2026.1783709 (PMC13043650; doi:10.3389/fcimb.2026.1783709)

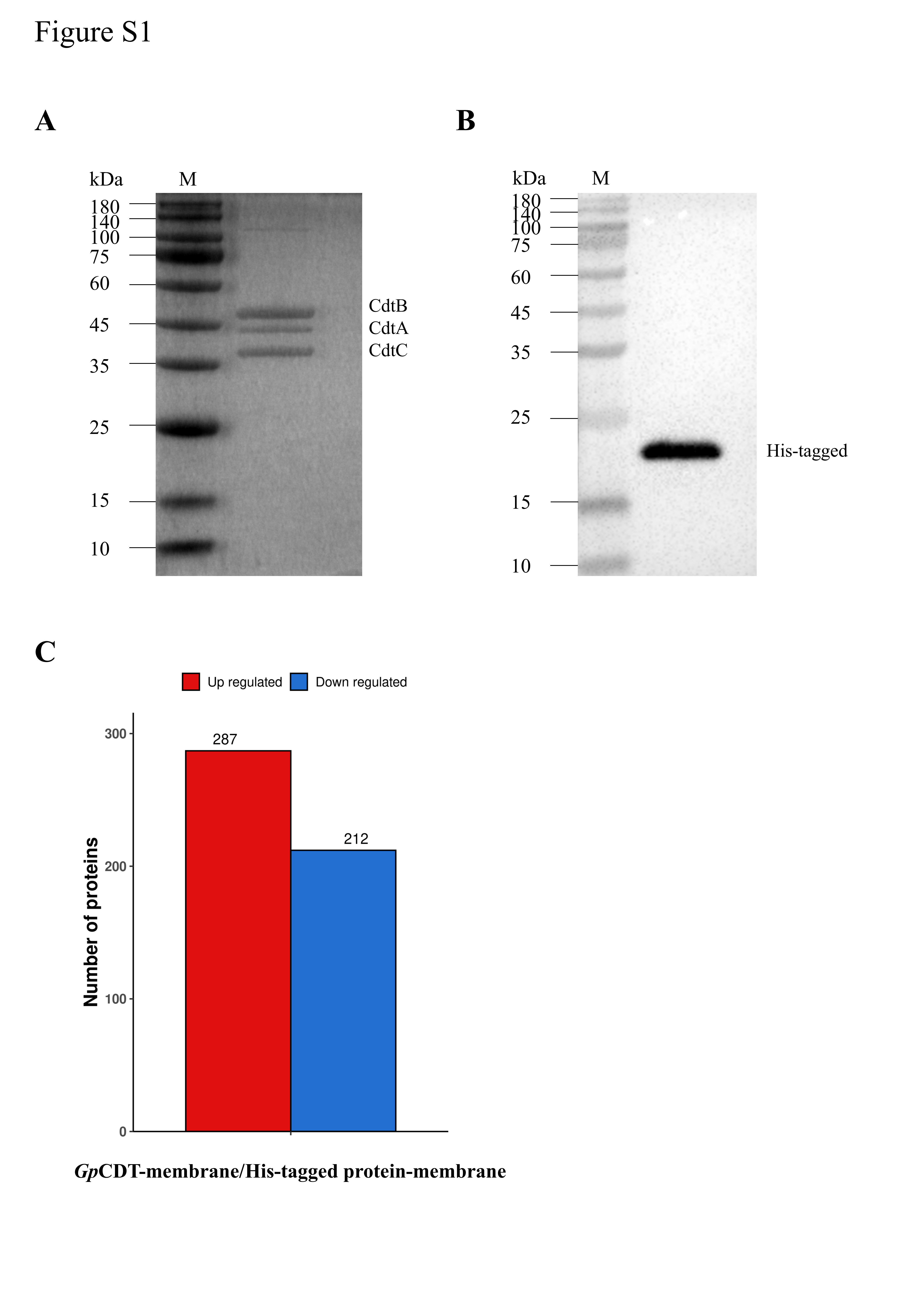

Supplement: Supplementary Figure 1 — Preparation of biological materials for LC-MS/MS analysis and identification of differentially expressed proteins. (A) SDS–PAGE analysis of purified GpCDT. (B) Western blotting of purified His-tagged protein. (C) Differentially regulated proteins identified by LC-MS/MS. [file Image1.tif]

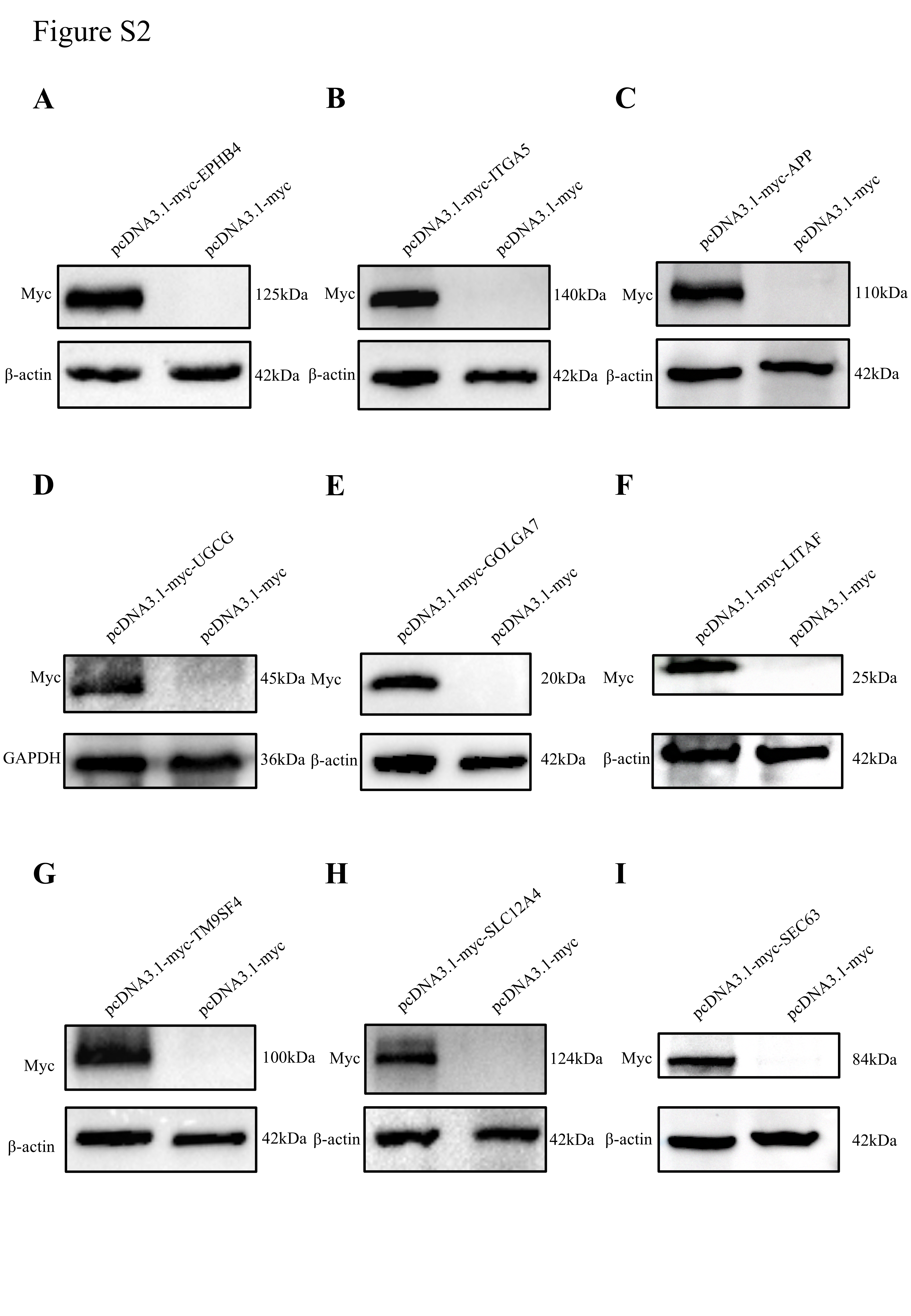

Supplement: Supplementary Figure 2 — Western blotting of 9 host cell proteins expression in HEK-293T Cells. (A-I) pcDNA3.1-Myc-EPHB4, pcDNA3.1-Myc-ITGA5, pcDNA3.1-Myc-APP, pcDNA3.1-Myc-UGCG, pcDNA3.1-Myc-GOLGA7, pcDNA3.1-Myc-LITAF, pcDNA3.1-Myc-TM9SF4, pcDNA3.1-Myc-SLC12A4, pcDNA3.1-Myc-SEC63 and pcDNA3.1-Myc Vector were transfected into HEK-293T for 48h. The eukaryotic expression of the protein was detected using rabbit anti-Myc-tag pAb. [file Image2.tif]

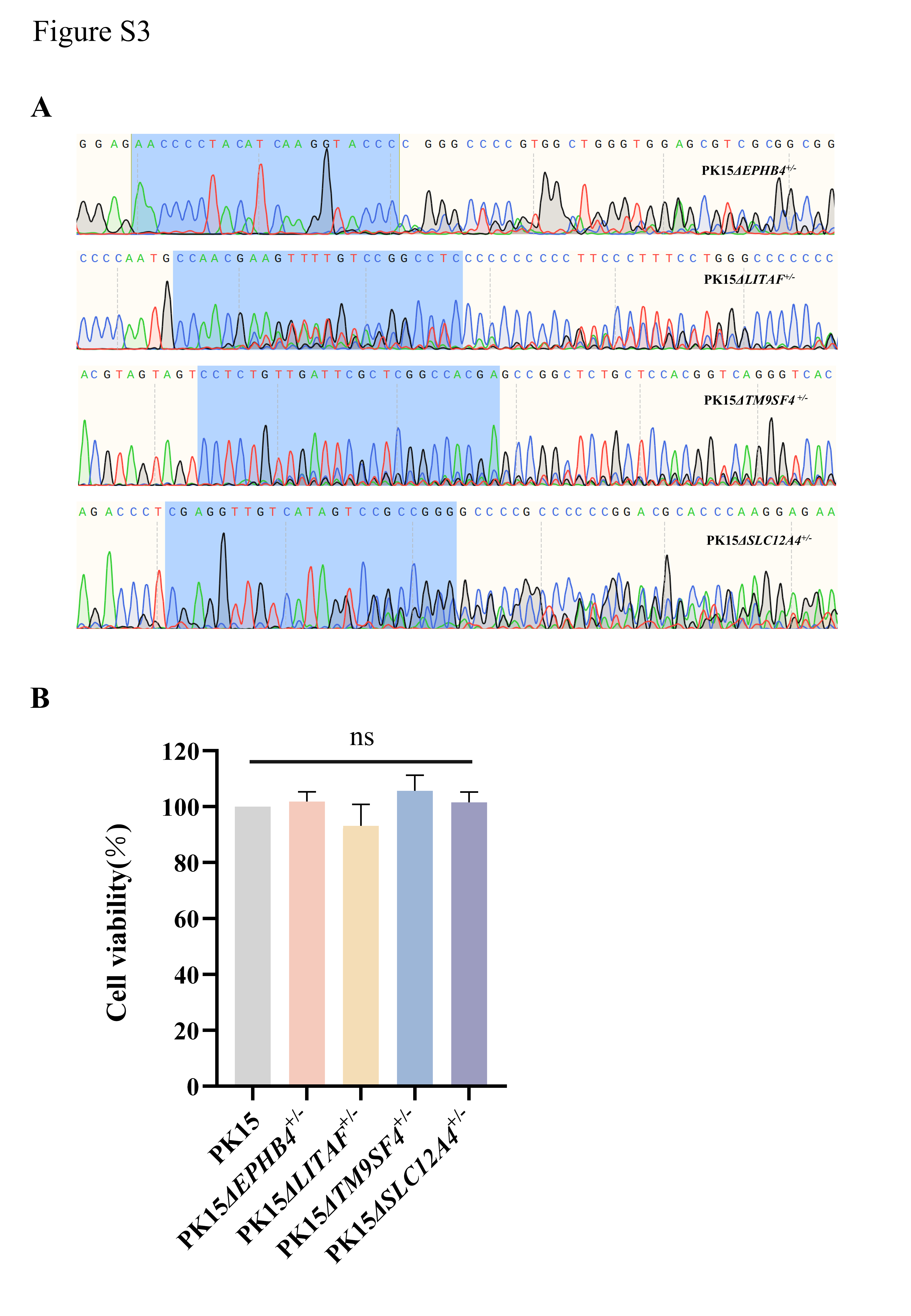

Supplement: Supplementary Figure 3 — Construction and validation of PK15 polyclonal gene knockout cell lines. (A) DNA sequencing analysis of EPHB4, LITAF, TM9SF4 and SLC12A4 polyclonal knockout PK15 cell lines. (B) The PK15 cell and polyclonal knockout PK15 cell lines were seeded into 96-well microplates and cultured for 48 h. Cell viability as determined by CCK-8 reagent at 450 nm. One-way analysis of variance (ANOVA) was employed for comparisons between groups. Data are shown as mean ± SD (n = 4), ns, p > 0.05. [file Image3.tif]

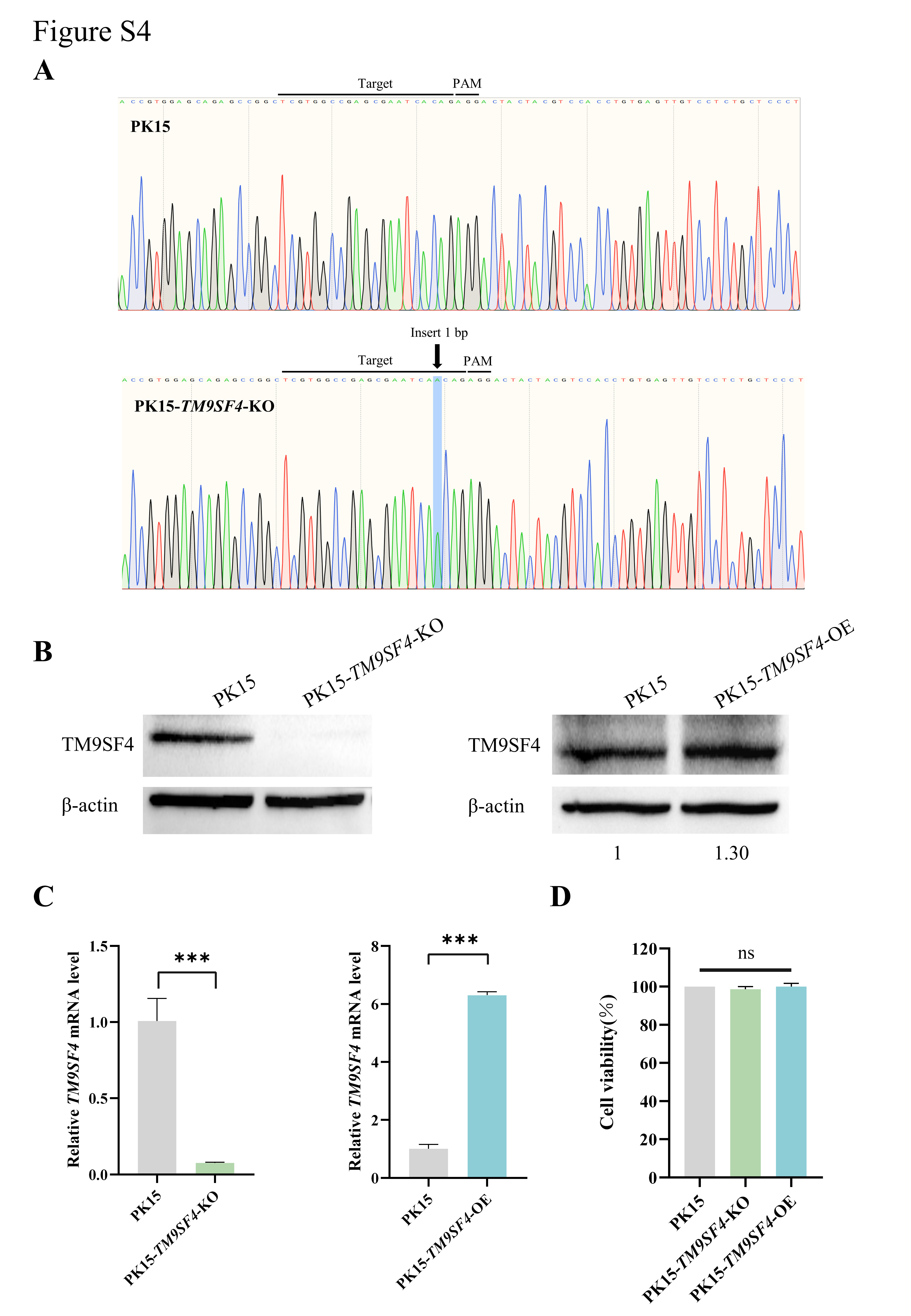

Supplement: Supplementary Figure 4 — Construction and validation of PK15-TM9SF4-KO and PK15-TM9SF4-OE. (A) Sequencing alignment analysis of TM9SF4 gene in PK15 and PK15-TM9SF4-KO. (B) Western blotting the TM9SF4 protein level in PK15, PK15-TM9SF4-KO and PK15-TM9SF4-OE. TM9SF4 levels were quantified by ImageJ. (C) qRT-PCR the relative TM9SF4 mRNA level in PK15, PK15-TM9SF4-KO and PK15-TM9SF4-OE. An unpaired Student’s t-test was employed for comparisons between groups. Data are shown as mean ± SD (n = 3), ***p < 0.001. (D) The PK15, PK15-TM9SF4-KO and PK15-TM9SF4-OE cell lines were seeded into 96-well plates and cultured for 48 h. Cell viability as determined by CCK-8 reagent at 450 nm. One-way analysis of variance (ANOVA) was employed for comparisons between groups. Data are shown as mean ± SD (n = 4), ns, p > 0.05. (KO: knockout; OE: overexpression). [file Image4.tif]
